# Osmotically balanced, large unilamellar liposomes that enable sustained bupivacaine release for prolonged pain relief in in vivo rat models

**DOI:** 10.1038/s41598-021-91624-2

**Published:** 2021-06-08

**Authors:** Hyebin Yoo, Jun Seok Park, Seung Soo Oh, Hyun Kang

**Affiliations:** 1 Department of Materials Science and Engineering, Pohang University of Science and Technology (POSTECH), Pohang, Republic of Korea; 2 Department of Surgery, Kyungpook National University Hospital, Kyungpook National University School of Medicine, Daegu, Korea; 3 Department of Anesthesiology and Pain Medicine, College of Medicine, Chung-Ang University, Seoul, Republic of Korea

**Keywords:** Biotechnology, Drug discovery

## Abstract

To efficiently prolong analgesic effects, we developed osmotically balanced, large unilamellar liposomes (~ 6 μm in diameter) in which highly concentrated bupivacaine (up to 30 mg/mL) was encapsulated, and their sustained bupivacaine release was highly effective in relieving postoperative pain over 24 h in a rat model. Our reverse-phase evaporation method based on non-toxic alcohol, ethanol, enabled simple and cost-effective production of bupivacaine-loaded liposomes, of which osmotic pressure was readily balanced to improve the structural stability of the enlarged unilamellar liposomes along with extension of their shelf life (> a month). The in vitro release profile verified that the release duration of the bupivacaine-loaded liposomes extended up to 6 days. For the in vivo study, male Sprague–Dawley rats were used for the incisional pain model, simulating postoperative pain, and the mechanical withdrawal threshold (MWT) was measured using a von Frey filament. Compared to the control group that received intraplantar administration of normal saline, the group of liposomal bupivacaine showed that the initially increased MWT gradually decreased up to 24 h, and importantly, the analgesic effect of the liposomal bupivacaine was maintained 6 times longer than that of bupivacaine only, proving the potential of effective long-acting anesthetics.

## Introduction

The management of acute postoperative pain is a crucial issue for clinicians. This is because poorly controlled postoperative pain increases postoperative complications, hospital stay, and financial cost and decreases patient satisfaction^1^. To address this issue, various modalities have been developed and conducted for postoperative pain control that can reduce the associated complications and enhance rehabilitation and quality of life after surgery^2^. However, the modalities to minimize the postoperative pain could cause medication-, instrument-, or technique-induced adverse effects. Furthermore, most patients who undergo surgery still experience considerable postoperative pain^3^, and unfortunately, the mechanisms of postoperative pain remain unclear^4^.

Currently, a multimodal analgesic regimen, using various kinds of medications and techniques, is recommended for better postoperative pain management and has become an integral component of perioperative pain management. Among these regimens, local anesthetics have gained popularity because of their convenient application, safety, and low cost. Intravenous lidocaine infusion^1^, incisional local anesthetic injection^5,6^, and intraperitoneal instillation of local anesthetics^7^ have efficiently decreased postoperative pain, nausea and vomiting, and opioid consumption. Importantly, these procedures have theoretical benefits; the decrease in pain intensity during surgery reduces the use of general anesthetics and the associated complications and hospital stay and increases patient satisfaction.

Bupivacaine is one of the most commonly used local anesthetics. It inhibits the voltage-gated sodium channels and NMDA receptors and has the longest duration of action among local anesthetics approved by the U.S. Food and Drug Administration (U.S FDA)^8^. However, after surgery, the pain extends beyond the analgesic duration of bupivacaine (2–4 h without epinephrine). This is a critical disadvantage of bupivacaine for perioperative pain control in clinical settings. Various methods, such as applying adjuvants (including dexamethasone and dexmedetomidine) and sustained release of local anesthetic, have been proposed and attempted to prolong the analgesic duration. Among these methods, injectable liposomal suspension that encapsulates bupivacaine, called EXPAREL, has been proven to extend the acting duration of bupivacaine and approved for clinical use by the U.S FDA. However, EXPAREL is produced by a complicated double emulsification process, and expensive neutral lipids (e.g., triglyceride) must be included in the lipid component for successful EXPAREL formation. Therefore, a simpler and cheaper process is required to develop lipid-based, long-acting bupivacaine to overcome these drawbacks.

As an alternative, we can readily use unilamellar liposomes for long-acting anesthetics, and the liposomal structures can be easily produced by a simple and cost-effective process, suitable for mass production. Individual unilamellar liposomes with different characteristics (e.g., lipid compositions, drug contents, vesicle size, and even structural morphology) can also be developed and further assembled via post-treatments, which hold the potential for developing longer-acting anesthetics than typical multivesicular liposomes such as EXPAREL. Furthermore, the unilamellar liposomes can be highly useful for increasing the exposure of active drugs to local tissues when large amounts of drugs can be readily loaded. However, to date, only a few studies exist on the encapsulation of anesthetic drugs in unilamellar liposomes^9,10^; it encourages us to develop buipvaciane-loaded unilamellar liposomes of which performance can be comparable with that of complicatedly prepared, multivesicular liposomes.

**Figure 1:**
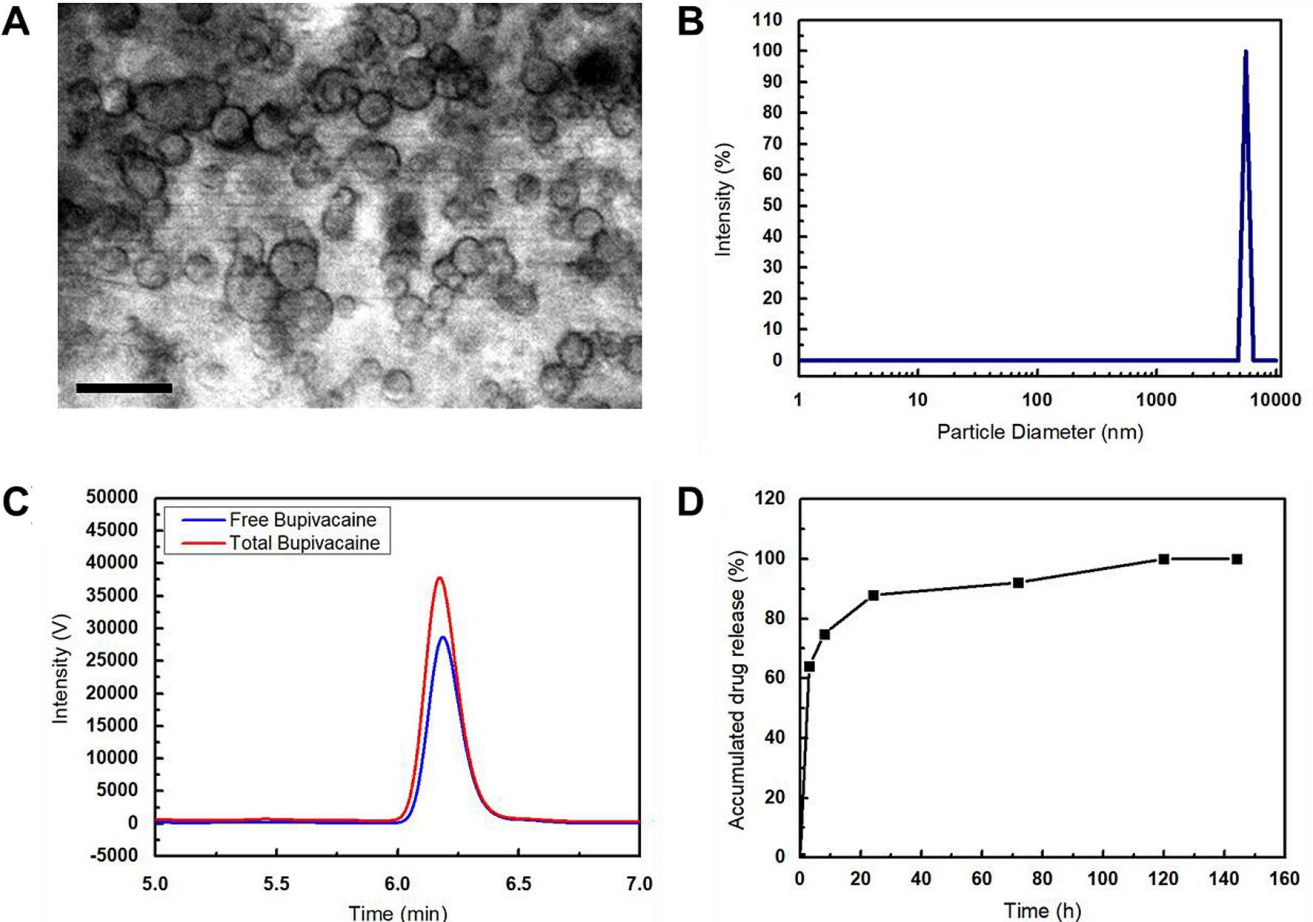
Characterization and in vitro pharmacokinetic study of bupivacaine-loaded liposomes. (**A**) Light microscope of bupivacaine-loaded liposomes at 500× magnification. Scale bar = 5 µm. (**B**) Particle size distribution of bupivacaine-loaded liposome suspension. (**C**) Chromatogram of free bupivacaine (blue) and total bupivacaine (red) for estimating encapsulation efficiency. The bupivacaine was monitored using UV detector at 262 nm. (**D**) In vitro release profile of bupivacaine-loaded liposomes.

This study demonstrates the development of highly concentrated bupivacaine-loaded unilamellar liposomes and their in vivo efficacy (Fig. 1). We successfully encapsulated highly concentrated bupivacaine (30 mg/mL), twice as high as the drug concentration in EXPAREL (15 mg/mL), in large unilamellar liposomes (~ 6 μm in in diameter). Importantly, by balancing the intra- and extra-vesicular osmolarity, the stability of the bupivacaine-loaded liposomes was fully secured, so the shelf life of our bupivacaine-loaded liposomes was significantly extended (> a month). However, when the osmotic pressure was imbalanced, the liposomal vesicles began to release bupivacaine, and the drug release was sustained up to 6 days. The production of EXPAREL relies on a complicated, two-step double-emulsion process requiring expensive resources including neutral lipids. On the other hand, our stable, yet large unilamellar liposomes can be produced by a neutral lipid-free, one-step reverse-phase evaporation method, exhibiting the potential for better economic efficiency. In an in vivo study, highly concentrated bupivacaine-loaded unilamellar liposomes showed the analgesic effect up to 24 h after surgery; compared to bupivacaine only, our liposomal suspension can be 6 times longer-acting anesthetics.

## Results

We successfully formulated large unilamellar vesicles containing a high concentration of bupivacaine, of which osmotic pressure was adjusted precisely to enhance stability for the sustained delivery of bupivacaine. Subsequent to the characterization of the bupivacaine-loaded liposomes, in vitro release behavior and in vivo efficacy after the application into rats were thoroughly investigated and compared with the solution that contained bupivacaine only. The results show that the bupivacaine duration was significantly prolonged up to 24 h, whereas that of the bupivacaine solution was much quicker. Importantly, liposomal bupivacaine showed a more enhanced analgesic effect than that with bupivacaine.

### Preparation and characterization of liposomal bupivacaine

We successfully prepared bupivacaine-loaded liposomes with high efficiency using a reverse-phase evaporation technique^11^. We employed reverse-phase evaporation, rather than thin-film hydration, to improve the efficiency of drug loading^11^, and ethanol was chosen as the organic solvent for liposome preparation, instead of the commonly used diethyl ether, because the residue of this biocompatible solvent within the resulting liposomes would not harm human health^3^. Specifically, we prepared a biphasic system by mixing a bupivacaine-containing aqueous phase and a lipid-dissolved organic phase. The mixture was then sonicated to form a homogeneous dispersion. After slow evaporation of organic solvents, a liposome-containing aqueous suspension was eventually generated. Unentrapped bupivacaine was washed with phosphate-buffered saline (PBS), and the bupivacaine-loaded liposomes were harvested via centrifugation. Balancing the osmotic pressures is crucial to improve the stability and shelf life of the formulated vesicles. Thus, the washed liposomes were resuspended in isotonic PBS. The final bupivacaine content in the liposome suspension was approximately 5 mg/mL, similar to commercial bupivacaine^12^.

We characterized our bupivacaine-loaded liposomes, and subsequently examined the in vitro drug release profile. Figure 1A shows the morphology of the prepared liposomes, which are spherical, with similar size distributions. Similar to the optical microscopy observations, the dynamic light scattering measurements confirmed that our liposomes were of the same size, yielding a single narrow peak centered at a mean particle size of 5560 nm (Fig. 1B). We calculated the drug encapsulation efficiency (Fig. 1C) using high-performance liquid chromatography (HPLC) and quantified the unentrapped bupivacaine (blue) and total bupivacaine (red). The maximum encapsulation efficiency was determined from three different batches to be 25.8% (Table 1). With osmotic balance, the bupivacaine-loaded liposomes could be stored at 4 °C over a month; they maintained structural integrity with minimal bupivacaine leakage compared to the osmotically imbalanced liposomes (Figure S1). Figure 1D shows the in vitro release profile of bupivacaine from the drug-loaded liposomes. Within 24 h, 87.9% of the bupivacaine was detected to be released from the liposomes. Before reaching equilibrium, the release duration from the bupivacaine-loaded liposomes was extended up to 6 days.

**Table 1:** Entrapment efficiency of bupivacaine-loaded liposomes.

Batch number	Entrapment efficiency (%)
1	25.8
2	24.2
3	20.1

### In vivo application of liposomal bupivacaine

All the selected rats completed the present study. The rats remained well-groomed and appeared to ingest a normal amount of food and water throughout the experimental period. Except for the impaired weight-bearing on the area of the incision, the gait appeared unaffected. None of the rats had complications of the surgical wound.

**Figure 2:**
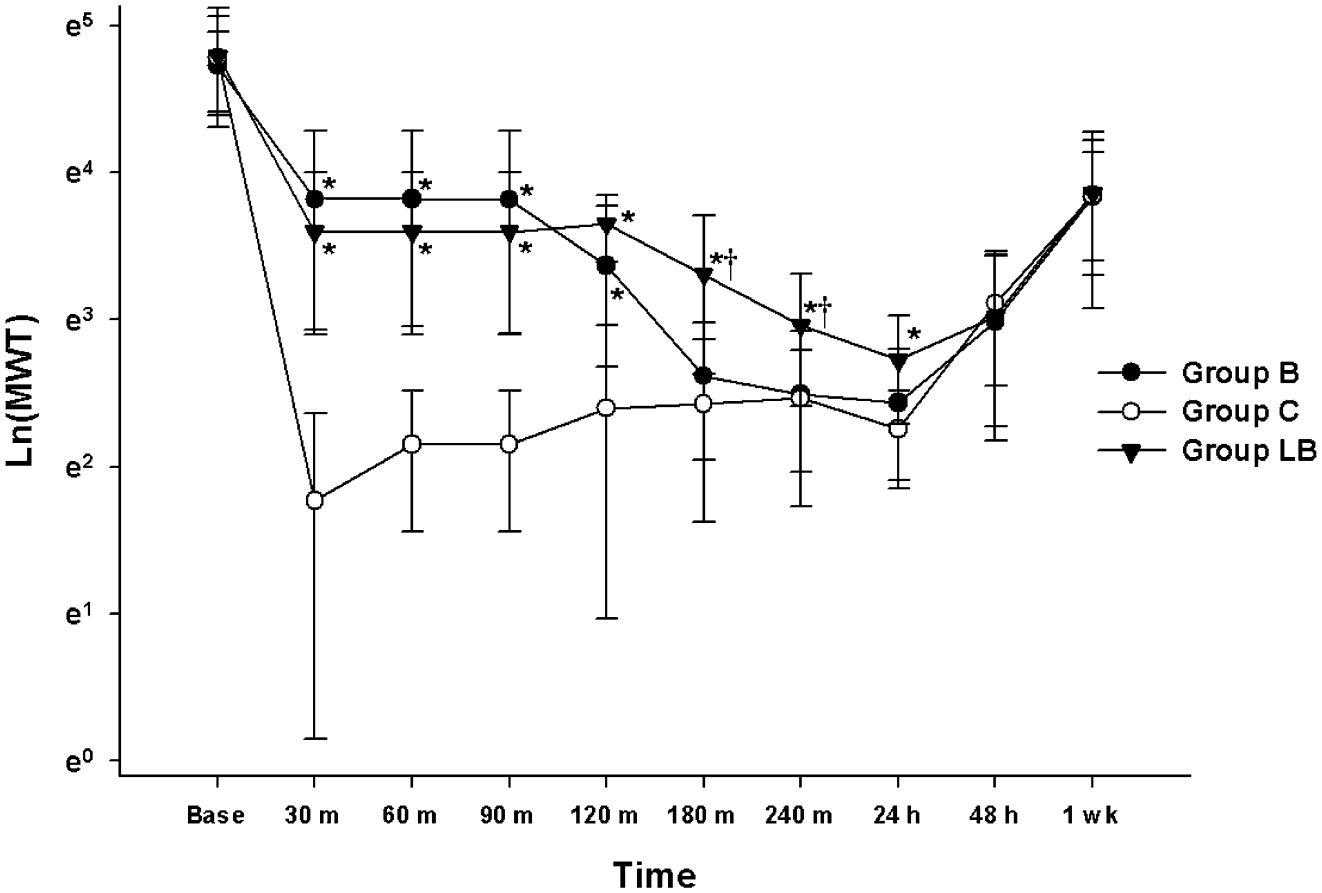
Evaluation of analgesic effects of bupivacaine and liposomal bupivacaine. The graphs are presented as mean ± standard deviation of the mechanical withdrawal threshold with von Frey filaments. Group B: received intra-plantar bupivacaine, Group C: received intra-plantar normal saline, Group LB: received intra-plantar liposomal bupivacaine, Base: baseline, MWT: mechanical withdrawal threshold. *P < 0.05 compared with the control group, † *P* < 0.05 compared with the bupivacaine group.

Figure 2 shows the MWT changes measured at base, 30, 60, 90, 120, 180, 240 min, 24 h, 48 h, and one week from the surgery. The results of the MANOVA test show a statistically significant difference between the groups (F[20.00, 48.00] = 4.282, *P* < 0.001: Wilk’s lambda = 0.129, partial η^2^ = 0.641). The MWT at the base was not significantly different among the groups. However, the MWT at 30, 60, 90, 120, 180, 240 min, and 24 h in the group with liposomal bupivacaine (group LB) and that at 30, 60, 90, and 120 min in the group with bupivacaine (group B) were significantly higher than that of the control group with normal saline (group C). The MWT at 180 and 240 min in group LB was significantly higher than that of group B. The linear mixed-effects model (LMEM) showed significant differences between the groups (F[2, 236.51] = 20.90, *P* < 0.001). Significant differences were observed between groups B and LB and group C (estimated difference in means (MD): 0.25 [0.11 to 0.39] and *p* < 0.001; MD: 0.45 [0.31 to 0.59] and *p* < 0.001, respectively).

## Discussion

Skin incision and tissue injuries from surgical procedures induce central neuronal excitability, leading to peripheral and central sensitization^13^, which can develop into chronic postoperative pain^14^. Postoperative pain control still depends on opioids, which have limited use owing to their adverse effects, including postoperative nausea and vomiting, respiratory depression, and urinary retention. Several strategies have been investigated for the prevention and treatment of postoperative pain. However, most patients undergoing surgery still experience considerable postoperative pain.

A nerve block and the direct application to the surgical site using local anesthetics have gained popularity because of convenience, safety, and low cost^5^. They also interrupt the conduction of pain signals to the brain, decreasing the possibilities of hyperalgesia and allodynia^15^. However, the analgesic duration of traditional long-acting local anesthetics has been insufficient, limiting these benefits. Furthermore, high-dose local anesthetics sometimes cause cardiovascular and central nervous system toxicity when their concentrations are maintained in the body^16^.

One of liposomal bupivacaine formulation, namely EXPAREL, wherein bupivacaine is encapsulated in multivesicular liposomes for sustained release, was approved for use as an injection in surgery in 2011 and proposed to provide extended postoperative analgesia, up to 72 h^17^. Several studies have evidenced the efficacy and safety of liposomal bupivacaine for wound infiltration^18,19^ and as field^20,21^ and nerve blocks^22^. However, controversies exist regarding its analgesic efficacy^1,23^. Many studies have failed to detect the differences in analgesic effects between liposomal bupivacaine and traditional long-term local anesthetics, especially at the 24 h mark after surgery^24,25^. Thus, this study aimed to explore the analgesic effect of the developed liposomal bupivacaine in detail.

Stable unilamellar liposomes containing a high concentration of bupivacaine were prepared through modified reverse-phase evaporation using ethanol. This study shows that even simple unilamellar liposomes can serve as sufficient drug carriers for prolonged delivery if their morphology, drug concentration, and osmotic pressure are well-engineered. In the in vitro study, the release duration of our bupivacaine-loaded liposomes was extended up to 6 days.

In the in vivo study, we demonstrated that our liposomal bupivacaine (Group LB) reduced postoperative pain for up to 24 h after surgery, longer than in the case of only bupivacaine (Group B), of which analgesic effect was maintained up to 240 min. Moreover, the liposomal bupivacaine (Group LB) showed a better analgesic effect at 180 min and 240 min after surgery than in the case of bupivacaine (Group B). Complications related to local anesthetic toxicity or mortality were not observed during the behavioral test and observational period. This enhanced analgesic effect is mainly due to the well-engineered characteristics of liposomal bupivacaine. The large size of the unilamellar liposomes (~ 6 μm in in diameter) have enabled the avoidance of absorption into the lymph or capillary network, and the high concentration of the encapsulated drug (30 mg/mL) afforded the delivery of large quantities of active bupivacaine to the local administration site. The osmotically balanced buffer condition may also have helped to improve the stability and half-life of the liposomal drugs.

In the in vivo study, an incisional pain rat model developed to reflect and simulate human postoperative conditions was used^13^, where peak time, intensity, and pain duration were similar to those in the case of human postoperative pain. Surgical incision and intraoperative injury may accentuate central neuronal excitability, leading to peripheral and central sensitization^26^ and, finally, chronic postoperative pain. A rat model of incisional pain is easy to apply to this study as it includes an incision followed by simple procedures and has shown reproducibility and reliability in several experiments^13^. Thus, it is widely used and accepted in the field of pain research.

Despite the above-mentioned results, this study has certain limitations. The study only evaluated the effect of liposomal bupivacaine over a short duration. A longer period of MWT evaluation is necessary to evaluate the effect of liposomal bupivacaine on chronic pain. Plasma bupivacaine concentration was not monitored in an animal study. This may help clarify the in vivo pharmacokinetics and pharmacodynamics of liposomal bupivacaine.

We anticipate that the performance of the prepared liposomal bupivacaine may be further improved through additional structural engineering. If the unilamellar liposomes can be assembled via post-treatment, interconnected structures of numerous aqueous chambers containing drugs will be generated, resulting in a morphological structure similar to that of multivesicular liposomes such as EXPAREL. These easily formed, closely packed, non-concentric vesicles would offer a novel approach to sustained-release drug delivery by staying at the administration site as a drug depot for a long period.

This study successfully demonstrated the sustained bupivacaine release using large, yet stable, unilamellar liposomes. Osmotic balancing of the liposomal structures enabled the encapsulation of highly concentrated bupivacaine (up to 30 mg/mL) into the enlarged unilamellar liposomes, the shelf life of which is increased by over a month. The intraplantar administration enabled the sustained release of bupivcaine over 24 h (Group LB). The analgesic effect was successfully validated in a rat model, wherein the analgesic duration was 6 times higher than that when only bupivacaine was used.

## Methods

### Preparation and characterization of liposomal bupivacaine

**Figure 3:**
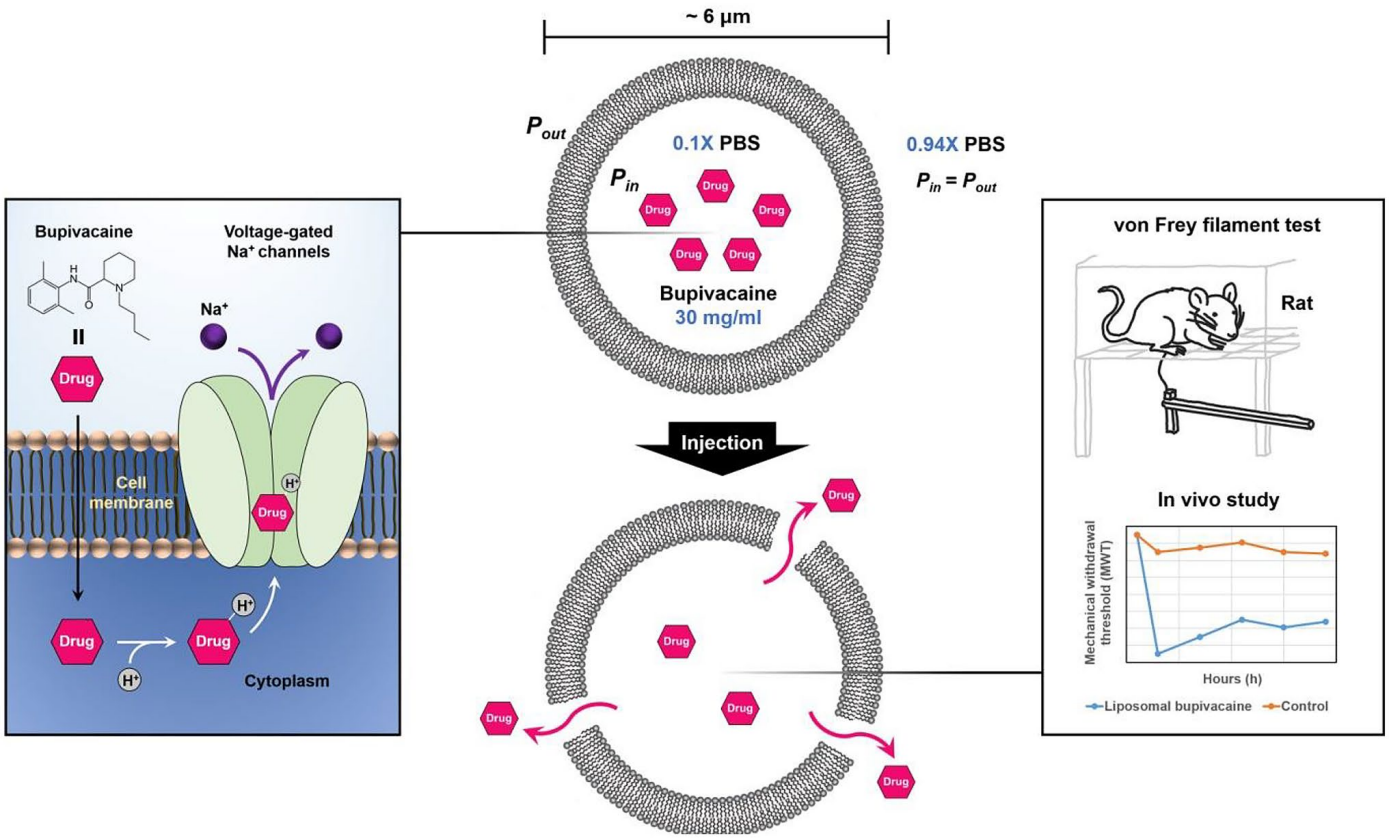
Schematic representation of osmotically balanced, large unilamellar liposomes (~ 6 μm in diameter) in which highly concentrated bupivacaine (up to 30 mg/mL) was encapsulated for sustained bupivacaine release and subsequent prolonged pain relief. Bupivacaine binds to the voltage-gated sodium channels and blocks sodium influx, preventing the conduction of pain signals. To evaluate the analgesic duration of liposomal bupivacaine in vivo, male Sprague–Dawley rats were used to measure the mechanical withdrawal threshold (MWT) with von Frey filaments.

The overall study processes are presented in Fig. 3.

#### Materials

1,2-Dioleoyl-sn-glycero-3-phosphocholine (DOPC), cholesterol, and bupivacaine hydrochloride were purchased from Sigma (St Louis, MO, USA), and 1,2-dipalmitoyl-sn-glycero-3-phosphoglycerol (DPPG) was purchased from Cayman (Ann Arbor, MI, USA).

#### Preparation of bupivacaine liposomes

The bupivacaine liposomes were prepared based on the reverse-phase evaporation methods developed by Szoska *et* al. 100 mg of DOPC, 100 mg of cholesterol, and 20 mg of DPPG were dissolved in 20 mL of chloroform/ethanol (1:1, v/v) in a 100 mL round-bottom flask. 5 mL of bupivacaine-HCl solution (0.1X PBS, 30 mg/mL bupivacaine-HCl) was added to this solution. The resulting two-phase system was sonicated briefly for 5 min in a bath-type sonicator. The ethanol and chloroform were removed at 40 °C via rotary evaporation under reduced pressure to form the final liposome aqueous dispersion. The liposomes were washed with 0.94 × phosphate-buffered saline (PBS) and harvested via centrifugation at 2000 g to separate the free drug from the vesicles. After washing, the liposomes were resuspended in 4 mL of 0.94 × PBS to yield a liposome suspension with an approximate bupivacaine concentration of 5 mg/mL.

#### Characterization of bupivacaine liposomes

The morphology of the bupivacaine liposomes was estimated using an optical microscope (Leica DM 4000 M). The particle size was measured using a particle size analyzer (Malvern Zetasizer).

#### Determination of encapsulation efficiency

The drug encapsulation efficiency was determined by comparing the amount of the encapsulated bupivacaine (D_en_) with the total amount of bupivacaine in the preparations (D_tot_). A total of 0.1 mL of the liposome suspension was dissolved in 10 mL of the extraction solution (0.2% Triton X-100, 28% ethanol, 71.8% water, v/v) to extract the bupivacaine from the liposome. The total drug content (D_tot_) in the suspension was quantified using HPLC. The free drug (D_free_) was separated from the pellet via centrifugation (2000 × g, 10 min) and quantified using HPLC. The encapsulation efficiency was estimated using the following equation:

1 $${\text{En}}\left( {\text{\% }} \right) = \frac{{\left( {{\text{Dtot}} - {\text{Dfree}}} \right)}}{{{\text{Dtot}} \times 100}}$$

#### In vitro release

The in vitro drug release of the bupivacaine liposome was determined using dialysis bags (MW cutoff 10 K). Two milliliters of the liposome suspension was transferred to the dialysis bag placed in a flask containing 200 mL of 0.94 $\times$ PBS. The flasks were incubated at 37 °C under constant stirring. Samples were collected at time points of 3, 8, 24, 72, 120, and 144 h and analyzed using HPLC.

### In vivo application of liposomal bupivacaine

The experimental protocols were reviewed and approved by the Institutional Animal Care and Use Committee at Chung-Ang University (2020–00,131). All the experiments were conducted following the guidelines established by the National Institutes of Health, the policies of the International Association for the Study of Pain for the Use of Laboratory Animals, and the guidelines recommended in the Animal Research Reporting In Vivo Experiments (ARRIVE) statement^27^.

#### Animal preparation

Adult male Sprague–Dawley rats weighing 250–300 g (Coretec Laboratories, Seoul, Korea) were used in this study. The rats were habituated in a colony room for a week before the experimental study. Two rats were housed in each cage at 22 ± 0.5 °C with a 12:12 h light–dark cycle. Food and water were available ad libitum. Female rats were not included in this study to avoid the impact of hormonal fluctuations on the pain threshold^28^. Rats with any abnormalities were excluded.

#### Group allocation and blindness

To assess the analgesic effects of liposomal bupivacaine and bupivacaine, the rats were randomly divided into one of three groups: Group B, Group C and Group LB. The rats in Group B, Group C and Group LB received intra-plantar bupivacaine, normal saline and liposomal bupivacaine, respectively.

Random assignment was based on a table generated by a computer-applied Wei’s Urn model using PASS™ 11 software (NCSS, Kaysville, UT, USA). The randomization code was generated by a statistician, not otherwise involved in the study.

For allocation concealment, another investigator, not involved in this study, prepared the syringes containing the study drugs for the experiments. For intra-plantar application, 1 mL syringes containing 0.2 mL of normal saline or study drugs were prepared. The syringes were covered with opaque tape and numbered sequentially according to a randomized list of the respective experiments. The prepared syringes were delivered to the researcher in charge of the surgery, who participated only in the surgery and was blind to the group assignment.

#### Surgical procedure

**Figure 4:**
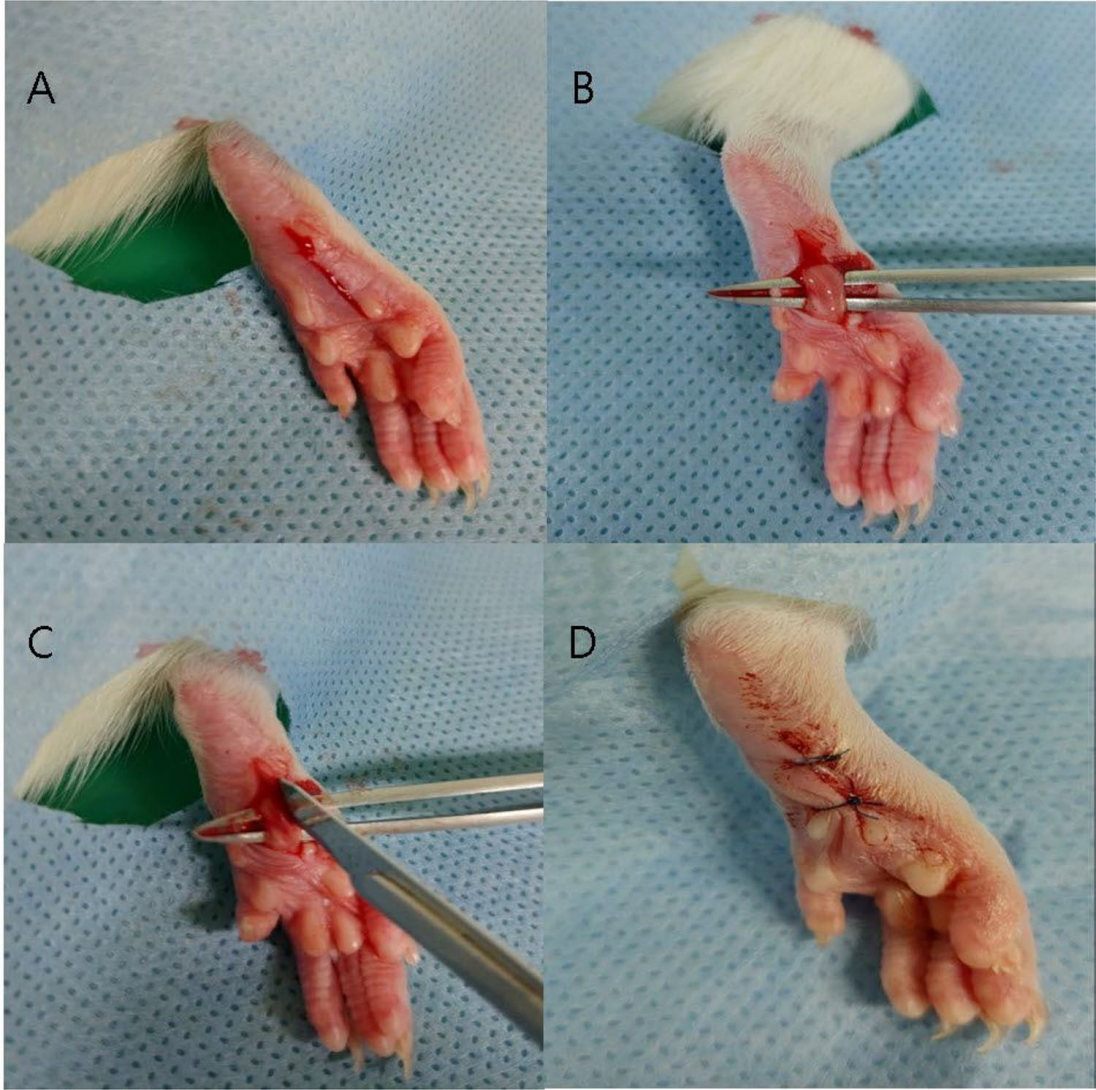
Incisional pain model preparation. (**A**) Skin incision was made on the plantar area of the hind paw. (**B**) The plantaris muscle was elevated. (**C**) An incision was made longitudinally, keeping the muscle origin and insertion site intact. (**D**) The incision was closed with two interrupted horizontal mattress sutures.

All surgical procedures were performed under sterile conditions. The rats received general anesthesia as an anesthetic, induced with 6% isoflurane in 100% oxygen inside a sealed clear plastic chamber until the rats became immobile. It was then maintained on a non-rebreathing anesthetic circuit mask using 1% to 3% isoflurane in 100% oxygen until the end of the surgery to prevent the rats from suffering during the surgical procedure. Before incision, cefazolin (20 mg/kg; Chong Kun Dang Pharmaceutical Co., Korea) was administered subcutaneously. The plantar surface of the left hind paw of each rat was prepared aseptically for surgery. The incisional pain model was created, as previously described^13^, with minor modifications in the reported technique. At a point, approximately 0.5 cm distal to the tibiotarsal joint on the plantar surface of the left hind paw, a 1 cm longitudinal skin incision, extending towards the digits, was made with a blade (Fig. 4A). The plantaris muscle was isolated, elevated slightly, and incised longitudinally (Fig. 4B,C). Study drugs in the prepared syringes were administered to the incision site over the desiccated area. The incision was closed with two interrupted horizontal mattress sutures of 5–0 nylon (Fig. 4D) All the rats were allowed to recover, and their sutures were removed on the third postoperative day.

#### Sample size calculation

The primary outcome measured was the threshold of withdrawal by mechanical stimuli using von Frey filaments. A pilot study was conducted to measure the MWT in six incisional pain model rats (Group C) to estimate the group size for the study assessing the antinociceptive activity of bupivacaine (Group B) and liposomal bupivacaine (Group LB). The averages of the natural log-transformed MWT at the base, 30 min, 60 min, 90 min, 120 min, 180 min, 240 min, 24 h, 48 h, and one week after surgery were 4.68, 1.49, 2.05, 2.05, 2.04, 2.52, 2.49, 2.41 3.04, and 3.70 ln(mN), respectively. The standard deviations of the natural log-transformed MWT ranged from 0.06 to 0.89, and the autocorrelation between adjacent measurements on the same individual was 0.7. For the power calculations, we assumed that a first-order autocorrelation adequately represented the autocorrelation pattern. The Geisser-Greenhouse Corrected F test for the repeated measures of ANOVA was used to analyze the differences between groups. We aimed to detect increments of 50% and 60% in MWT in groups B and LB over group C. It was determined that, with α = 0.05 and power = 80%, ten rats were required per group. Considering a 20% follow-up loss, we utilized a total of 36 rats in the study.

#### Statistical analysis

The Shapiro–Wilk test was used to test the normality of variables. As MWT did not pass the Shapiro–Wilk test, natural log-transformation was tested^29^. The natural log-transformed variables passed the Shapiro–Wilk test. Therefore, we assumed that the normal distribution assumption for the parametric test was not violated and decided to apply repeated measures ANOVA**:** the within-subjects factors of times (at the base, 30 min, 60 min, 90 min, 120 min, 180 min, 240 min, 24 h, 48 h, and one week after surgery) and between-subjects factor of groups (group C, group B and group LB). As Mauchly’s sphericity test indicated that the assumption of sphericity was violated in the MWT test (χ^2^ (44) = 179.32, *P* < 0.001, Mauchly’s W = 0.002), we used one-way Wilk’s lambda multivariate analysis of variance (MANOVA): each group (group C, group B and group LB) and each time point (at the base, 30 min, 60 min, 90 min, 120 min, 180 min, 240 min, 24 h, 48 h, and one week after surgery) were independent factors, and the MWT at each time point (at the base, 30 min, 60 min, 90 min, 120 min, 180 min, 240 min, 24 h, 48 h, and one week after surgery) was the dependent variable. Univariate analysis of variance (ANOVA) using Bonferroni correction (α = 0.05/10 = 0.005) was used to compare the MWT at each time point.

The between-group differences for Ln(MWT) were analyzed using an LMEM, with time (at the base, 30 min, 60 min, 90 min, 120 min, 180 min, 240 min, 24 h, 48 h, and one week after surgery) and group as independent fixed factors and individual patients as random effects.

Individual measurements were expressed as the mean ± standard error and analyzed with SPSS 23.0 (IBM Corp., Armonk, NY, USA). A *P* value of 0.05 was considered statistically significant.

## Supplementary Information

Supplementary Information.
