# Supplementary material for: Osmotically balanced, large unilamellar liposomes that enable sustained bupivacaine release for prolonged pain relief in in vivo rat models
Source: Sci Rep. 2021 Jun 8;11:12096. doi: 10.1038/s41598-021-91624-2 (PMC8187397; doi:10.1038/s41598-021-91624-2)
Supplement: Supplementary file 1 — Supplementary Information. [file 41598_2021_91624_MOESM1_ESM.docx]

**Supplementary Information**

**Osmotically balanced, large unilamellar liposomes that enable sustained bupivacaine release for prolonged pain relief in in vivo rat models**

Hyebin Yoo,^1*^ Jun Seok Park,^2*^ Seung Soo Oh,^1†^ Hyun Kang^3†^

* equally contributed to this work

^1^ Department of Materials Science and Engineering, Pohang University of Science and Technology (POSTECH), Pohang, Republic of Korea.

^2^ Colorectal Cancer Center, Kyungpook National University Chilgok Hospital, Daegu, Republic of Korea.

^3^ Department of Anesthesiology and Pain Medicine, Chung-Ang University, college of medicine, Republic of Korea.

**^†^ Corresponding Author**

Hyun Kang: roman00@naver.com

Seung Soo Oh: [seungsoo@postech.ac.kr](mailto:seungsoo@postech.ac.kr)


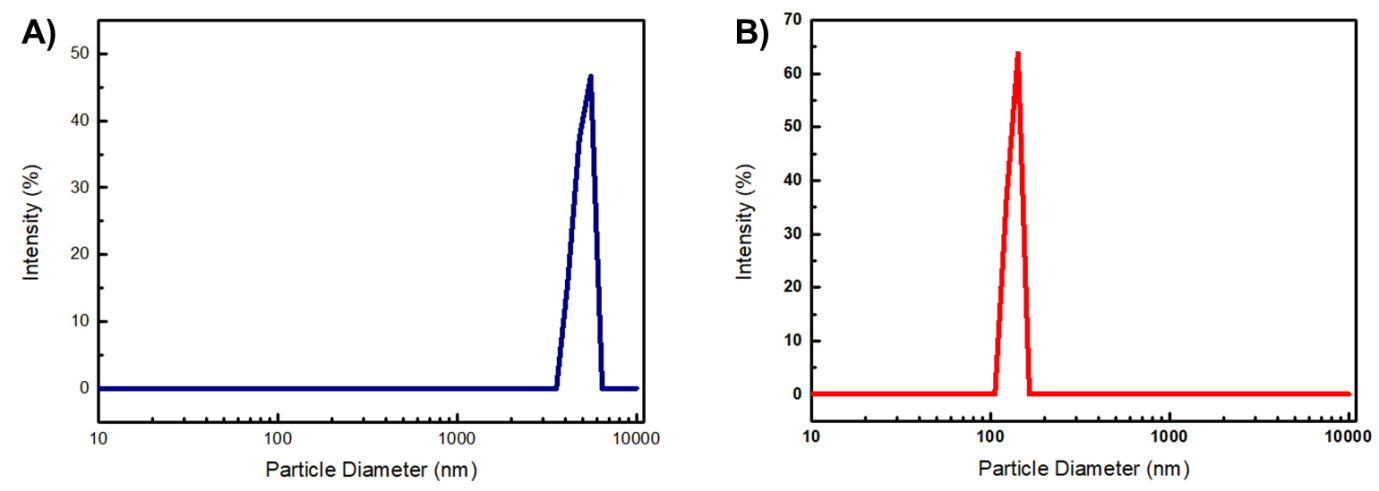


Figure S1. Particle size distribution of bupivacaine-loaded liposomes when stored at 4°C over a month. A) When the liposomes were osmotically balanced, the mean particle size was 5589 nm, which is quite similar to the initial particle size. B) However, without osmotic balance, the mean particle size dramatically decreased to 135 nm, confirming the destruction of liposomes.

**
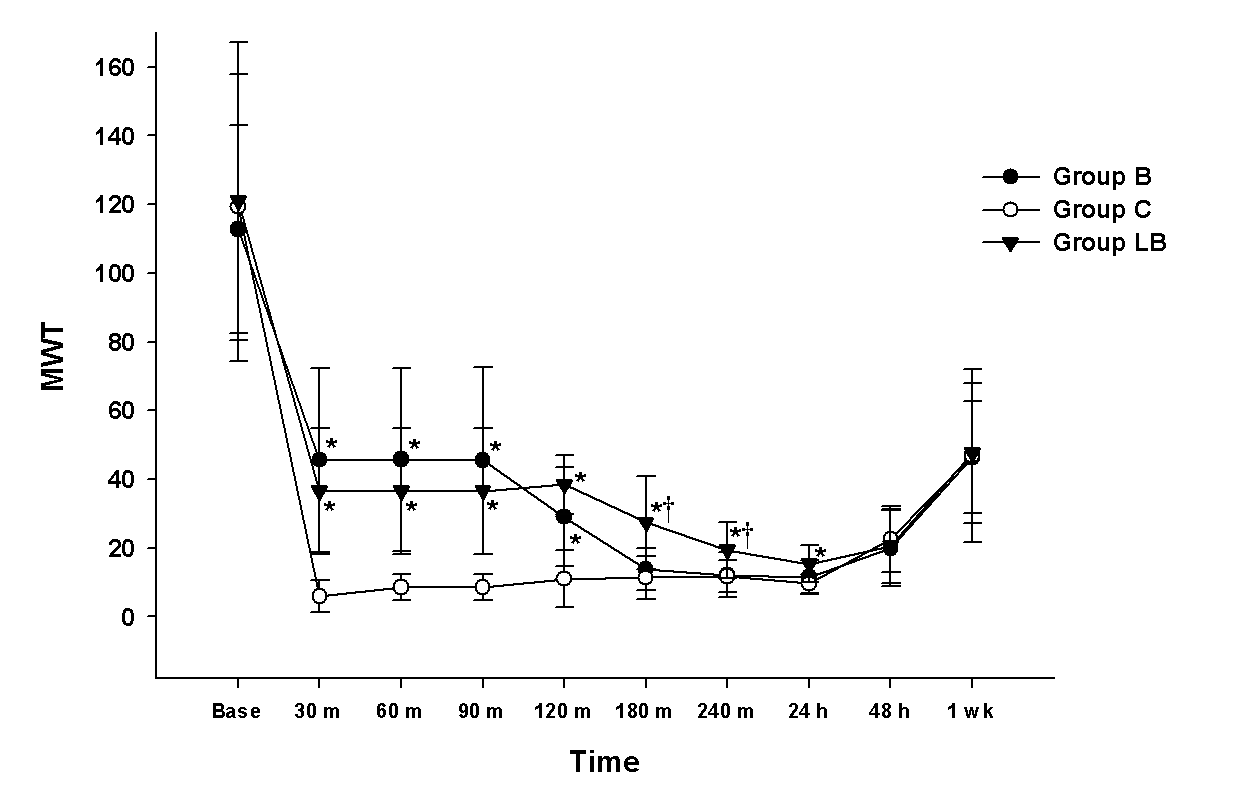
**

**Figure S2. Evaluation of analgesic effects of bupivacaine and liposomal bupivacaine.** The graphs are presented as mean ± standard deviation of mechanical withdrawal threshold with von Frey filaments. Group B: received intra-plantar bupivacaine, Group C: received intra-plantar normal saline, Group LB: received intra-plantar liposomal bupivacaine, Base: baseline, MWT: mechanical withdrawal threshold. *P <0.05 compared with the control group, † P<0.05 compared with the bupivacaine group.
